# LSTM-Based Virtual Load Sensor for Heavy-Duty Vehicles

**DOI:** 10.3390/s24010226

**Published:** 2023-12-30

**Authors:** Abdurrahman İşbitirici, Laura Giarré, Wen Xu, Paolo Falcone

**Affiliations:** 1 Department of Electrical, Electronic and Information Engineering, University of Bologna, 40126 Bologna, Italy; abdurrahm.isbitiric2@unibo.it; 2 Department of Engineering “Enzo Ferrari”, University of Modena and Reggio Emilia, 41125 Modena, Italy; falcone@unimore.it; 3 Volvo Trucks, 40508 Gothenburg, Sweden; wen.xu@volvo.com; 4 Mechatronics Group, Department of Electrical Engineering, Chalmers University of Technology, 41296 Gothenburg, Sweden

**Keywords:** long short-term memory, mass estimation, recurrent neural network

## Abstract

In this paper, a special recurrent neural network (RNN) called *Long Short-Term Memory (LSTM)* is used to design a virtual load sensor that estimates the mass of heavy vehicles. The estimation algorithm consists of a two-layer LSTM network. The network estimates vehicle mass based on vehicle speed, longitudinal acceleration, engine speed, engine torque, and accelerator pedal position. The network is trained and tested with a data set collected in a high-fidelity simulation environment called Truckmaker. The training data are generated in acceleration maneuvers across a range of speeds, while the test data are obtained by simulating the vehicle in the Worldwide harmonized Light vehicles Test Cycle (WLTC). Preliminary results show that, with the proposed approach, heavy-vehicle mass can be estimated as accurately as commercial load sensors across a range of load mass as wide as four tons.

## 1. Introduction

The accurate knowledge of the vehicle mass is important in all types of heavy vehicles for both *energy efficiency* and *safety*, as will be explained next. The operation of the powertrain in, e.g., automatic gear shifting can be optimized by algorithms relying on knowledge of the vehicle mass [1]. Such information can also be used to provide truck drivers with recommendations aiming to adapt their driving style to the current vehicle mass and road profile. Similar recommendations can help improve vehicle safety by, e.g., estimating the braking distance based on the current mass [2]. In buses, mass estimation can be used to estimate the number of passengers for various purposes, ranging from resource planning and bus scheduling to adjusting the air conditioning based on the number of passengers [3]. Mass information can be used to determine vehicle ordering within a platoon. For example, the heaviest truck might lead a platoon, as its braking distance is larger than that of the others, and it may cause rear-end collisions when following lighter vehicles at a close distance. On the other hand, heavier trucks should be placed at the platoon tail while traveling uphill, in order not to excessively limit other trucks’ speed. Mass information, along with inertia, could be effectively used by active safety systems to prevent rollover [4].

It must be pointed out that load sensors exist [5] that are reliable (same lifespan as the vehicle), affordable, and calibrated with fairly simple procedures. Moreover, there are inclinometer-based weighing sensors that can be used on inclined surfaces or uneven terrain [6]. Nevertheless, in truck and trailer combinations, the presence of such sensors depends on the trailer’s manufacturer. Hence, load sensors cannot be assumed to be available on every trailer. In such situations, estimating the vehicle mass, rather than measuring it with a sensor, is definitely preferable. Even in the presence of a load sensor, a mass estimation algorithm could be used to detect any sensor failure, malfunctioning, or need for recalibration.

One of the most common algorithms that is used for mass estimation is recursive least squares (RLS) [7,8,9,10,11,12]. RLS is also used to jointly estimate mass and road grade if road grade is not available. In [7], an RLS mass estimation algorithm updates the mass estimate according to a set of rules: the absolute value of the longitudinal acceleration is more than 0.1 m/s${}^{2}$, the engine torque percentage is between 40 and 90%, the lateral acceleration is less than 5 m/s${}^{2}$, and the absolute engine torque gradient is less than 1000 Nm/s. Finally, the mass estimate is updated only if the brake pedal is not pressed and if specific gears are engaged. Such rules are defined according to the vehicle operation, where the vehicle model that the RLS estimation algorithm is built upon is more accurate, and so is the mass estimate. In general, defining such rules might be impractical and time-consuming. The mass, drag coefficient, and rolling resistance are jointly estimated in [8] using an RLS estimation algorithm built upon the vehicle longitudinal dynamics. First, the road grade is estimated, and then the mass is estimated by filtering the engine torque, road grade, vehicle speed, and acceleration by a second-order Butterworth low-pass filter. In [9], an RLS mass estimation algorithm is fed with data extracted from the measured signals by a supervisory algorithm that searches for maneuvers dominated by inertial dynamics (e.g., acceleration maneuvers). An RLS with multiple forgetting factors is proposed in [10] for mass and road grade, respectively; a single forgetting factor for both has proven not to be beneficial. The work in [11] shows how a nonlinear road grade and mass estimator can be designed by initializing the estimates with the initial guesses of mass and road grade calculated by an adaptive least square while assuming they are both constant. The RLS mass estimator in [12] builds upon the longitudinal and roll vehicle dynamics.

Model-based (e.g., RLS) estimation methods are very convenient as they also provide a measure of the estimation accuracy depending on the measurement noise. On the other hand, the estimate is as accurate as the system model, and obtaining accurate system mathematical models can be a lengthy and expensive process. In fact, the RLS-based vehicle mass estimation algorithm available in the literature must be coupled with additional logic that enables the mass update in conditions where the vehicle model is accurate. Such logic can be difficult and impractical to be tuned.

*Learning-based* methods can also be used for mass estimation of heavy vehicles. In [13], the authors used a shallow neural network with the rectified linear unit (ReLU) activation function for mass and road grade estimation, while [14] proposes a Deep Neural Network (DNN) with 15 fully connected layers with the leaky ReLU activation function to estimate the trailer mass. The first layer has 140 neurons, and the number of neurons in each layer dwindles down by 10, except for the output layer, which has one neuron that outputs the mass estimate. The authors also used RNN as a comparison with DNN for mass estimation, and they found error percentages of 9% and 8.5%, respectively.

In [15], the mass of heavy-duty vehicles is estimated by using Gaussian Belief Propagation with k-nearest factors (kNF-GBP), such that the effect of noisy data is attenuated without resorting to any additional sensors. Vehicle velocity and acceleration, fuel consumption, engine load, speed, reference torque, and GPS location are used as measurements. Such an approach provides the mean and variance of the mass estimate. The work in [16] proposes a Random Forest method for mass estimation of passenger weight on e-scooters. The algorithm is used for classification rather than regression since there are limited data. In [17], RLS is combined with a machine learning approach by using fuzzy logic. The estimates from the RLS and ML methods are weighted based on longitudinal acceleration, acceleration rate, and vehicle speed. An additional weight is assigned to the previous mass estimate that is used when both the ML and RLS methods are not reliable. The ML approach relies on a two-layer neural network with 1024 and 256 neurons.

The automotive industry has also contributed to the development of vehicle mass estimators. In [18], an RLS mass estimator is built upon the longitudinal dynamics and the measurements of wheel speeds, engine torque, gearbox data, brake pressures, and longitudinal acceleration. The initial mass estimate is calculated based on the seat belt sensors and fuel level information. If one of the doors is opened, the RLS is reset. In [19], the RLS algorithm estimates the vehicle mass from variations in the vehicle center of gravity height, measured by a lidar. The work in [20] estimates the mass in three steps. When the engine starts, the last estimated vehicle mass is used. After a certain time or when the vehicle reaches a defined velocity, the mass is estimated based on the longitudinal dynamics if the vehicle is moving in a straight line. Then, when the vehicle reaches the set velocity a second time, such as when it accelerates and then decelerates, the mass is calculated based on the total force difference divided by the difference in the acceleration values when the vehicle reaches the set velocity the first and second times. The reason why this last step is used is to decrease the error due to the noise of longitudinal acceleration and vehicle speed measurements. In [21], the vehicle mass is estimated from inertial sensor measurements that measure the vertical acceleration. As the unladen mass of the truck is usually known, if the longitudinal and lateral accelerations are negligible, the load mass can be estimated from the vertical acceleration using an RLS approach. In [22], a mass estimation method is proposed for vehicle platoons. The mass difference between adjacent vehicles can be estimated based on the longitudinal dynamics and the information exchanged by the trucks, including torque, brake, and gap information.

Although DNNs could be used, in principle, to solve the considered mass estimation problem, RNNs better fit such applications as their memory can be harnessed to learn the longitudinal vehicle dynamics. In this study, a special type of RNN called a Long Short-Term Memory network, is used to estimate the mass of heavy-duty vehicles. LSTMs are chosen because their structure alleviates the gradient vanishing issues typical of classical RNNs [23]. LSTMs have been used in a variety of applications related to the modeling, estimation, and control of dynamical systems. An online learning algorithm for nonlinear regression based on LSTM by using a particle filter approach is presented in [24]. A modified LSTM is used in [25] to predict the output response of nonlinear systems. In [26], LSTM and Gated Recurrent Unit (GRU) methods are used to predict the vertical acceleration of a large-scale ship model. In [27], various LSTM networks are examined in order to detect anomalies.

This paper explores the use of Long Short-Term Memory networks for mass estimation in heavy vehicles. Our objective is to understand the necessary training effort, in terms of the training data set size and training time, to achieve a mass estimate that is as accurate as the measurement obtained from the available commercial load sensors [5]. Our choice of the type of RNN is motivated by the objective of achieving the target accuracy with networks that are as shallow as possible. Furthermore, LSTM networks, compared to standard RNNs, alleviate gradient vanishing issues.

The paper is structured as follows. In Section 2, the longitudinal vehicle dynamics model is presented. Section 3 gives information about the background of neural networks and the methodology. NNs are firstly described in Section 3.1 and then RNN is explained in Section 3.2. Then, LSTM is shown in Section 3.3. In Section 3.4, how data are used to train and test a neural network is described. In Section 4, we propose a data-driven mass estimation algorithm. In Section 5, how data are collected is explained. Experimental validation is performed in Section 6, and the results are discussed there as well. In Section 7, the conclusion and suggestions for future work are written.

## 2. Longitudinal Vehicle Dynamics

**Figure 1:**
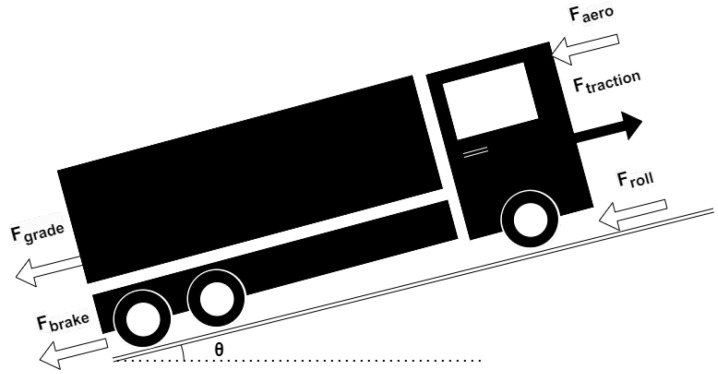
Longitudinal vehicle dynamics.

Before presenting the vehicle mass estimation algorithm, it is convenient to recall the longitudinal vehicle dynamics shown in Figure 1. The vehicle longitudinal velocity can be found as the solution of the differential equation

(1) $$\;m\dot{v}=F_{traction}-F_{brake}-F_{aero}-F_{roll}-F_{grade}.$$

In Equation (1), $F_{aero}$ is the air drag force, which can be modeled as in (2).

(2) $$\begin{array}{cc} F_{aero} & =0.5\rho c_{d}A_{f}{\left(v+v_{wind}\right)}^{2},\\ \; & =f_{aero}\left(\rho ,c_{d},A_{f},v,v_{wind}\right), \end{array}$$

where ρ is the air density, $c_{d}$ is the aerodynamic drag coefficient, $A_{f}$ is the area of the front side of the vehicle, and *v* and $v_{wind}$ are the speeds of the truck and the wind, respectively [28]. For the sake of simplicity, braking maneuvers are not considered in this paper. $F_{roll}$ is the rolling resistance force calculated as in (3).

(3) $$\begin{array}{cc} F_{roll} & =\mu mgcos(\theta ),\\ \; & =f_{roll}\left(\mu ,m,\theta \right), \end{array}$$

where θ is the road gradient, μ is the rolling resistance coefficient, *m* is the mass of the truck, and *g* is the gravitational acceleration. $F_{grade}$ is the force introduced by the road grade as in (4).

(4) $$\begin{array}{cc} F_{grade} & =mgsin(\theta ),\\ \; & =f_{grade}\left(m,\theta \right), \end{array}$$

and $F_{traction}$ is the traction or propulsive force (5) where

(5) $$\begin{array}{cc} F_{traction} & =\frac{T_{engine}-J_{eq}\dot{\omega }}{\frac{r_{wheel}}{g_{r}g_{final}}},\\ \; & =f_{traction}\left(T_{engine},J_{eq},\omega ,r_{wheel},g_{r},g_{final}\right), \end{array}$$

$T_{engine}$ is the engine torque, which can be estimated with various methods; $J_{eq}$ is the equivalent inertia; ω is the engine crankshaft speed; $g_{r}$ and $g_{final}$ are the gear ratio and final drive ratio, respectively; and $r_{wheel}$ is the radius of the wheel. From Equation (1), the vehicle mass *m* can be expressed as in (6).

(6) $$\begin{array}{cc} m & =\frac{F_{traction}-F_{brake}-F_{aero}-F_{roll}-F_{grade}}{\dot{v}},\\ \; & =f(u(t),\tau ), \end{array}$$

where u(t)= ($T_{engine}$, ω, θ, *v*, $\dot{v}$) is the input vector of the model (6), which includes the onboard sensors measurements, and $\tau =\left(\rho ,c_{d},A_{f},\mu ,g,J_{eq},r_{wheel},g_{r},g_{final}\right)$, which contains the physical parameters of the vehicle model. In principle, a mathematical model $\hat{f}$ could be built for the function *f* based on a vehicle mathematical model. In practice, such a function $\hat{f}$ rarely enables a sufficiently accurate estimate of the vehicle mass *m*. Hence, in this paper, we show how the function $\hat{f}$ can be built based on input (u(t)) and output (*m*) data samples.

## 3. Background on Artificial Neural Networks and Methodology

Consider a system

(7) y=f(u),

where u,y are the system input and output vectors, respectively. In Section 3.1, we recall how Artificial Neural Networks (ANNs) can be used to formulate a mathematical model

(8) $$\hat{y}=\hat{f}\left(x\right),$$

of system (7), starting from samples of the input and output, u,y, respectively. Note that, in general, the input vectors *u* and *x* of the functions *f* and $\hat{f}$, respectively, are different. Indeed, the input vector *x* may include all, or part of, the signals in *u* and additional signals as well. We then illustrate in Section 4 how NNs are used in this paper to design a mass estimation algorithm.

### 3.1. Neural Networks

**Figure 2:**
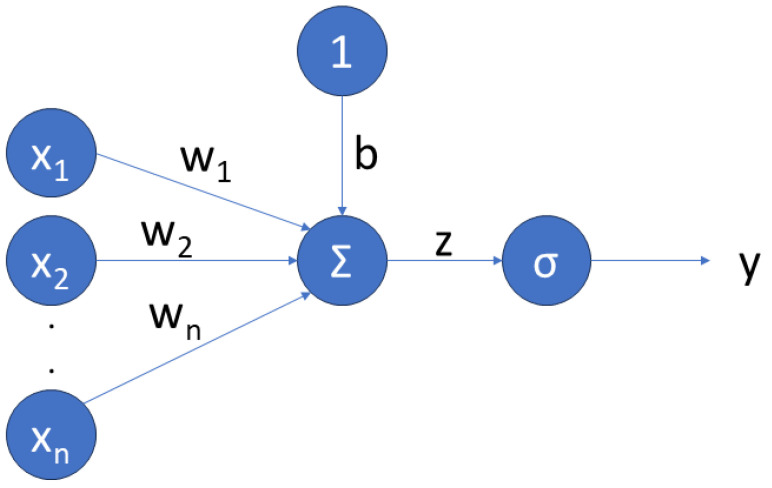
Perceptron.

ANNs are nonlinear functions, where a vector x(t) of inputs is mapped into an output $\hat{y}$. In the simplest ANN, which is called a *perceptron*, shown in Figure 2, all input signals are directly connected to an *output node*, which is called a *neuron* and maps the input to the output signals through an *activation function*. In an ANN, the output signal is expressed as

(9) $$\begin{array}{cc} \hat{y} & =\sigma \left(\sum_{i=1}^{n}w_{i}x_{i}+b\right),\\ \; & =\hat{f}\left(x,\;W,\;b\right), \end{array}$$

where $x\left(t\right)=\left[x_{1}\left(t\right),\ldots ,x_{n}\left(t\right)\right]$ is the input vector containing the *features* of the network, $W=\left[w_{1},\ldots ,w_{n}\right]$ and $b=\left[b_{1},\ldots ,b_{n}\right]$ are the network weights and biases, *n* is the number of inputs, $\hat{y}$ is the model output, and σ is the activation function. For the sake of readability, with a slight abuse of notation, the same symbol $\hat{f}$ as in (8) has been used in (9), although the two functions have different numbers of arguments.

**Figure 3:**
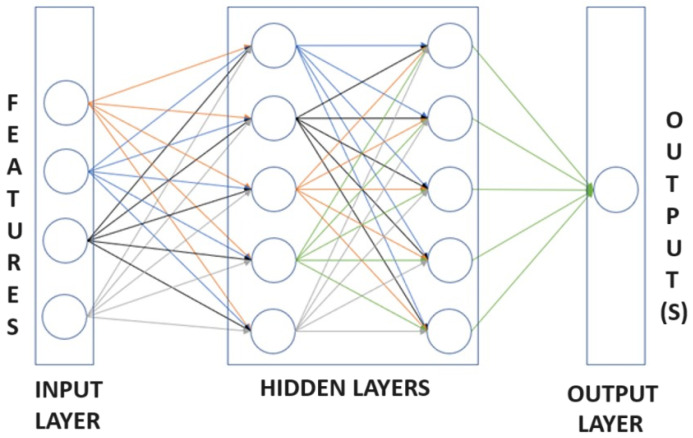
Feedforward neural network.

A network in which the inputs are mapped to the output with interconnected layers or neurons, rather than a single neuron, is called a feedforward neural network (FFNN) or Multilayer Perceptron (MLP), as shown in Figure 3. If a FFNN has two hidden layers or more, this is called Deep Neural Network (DNN).

**Figure 4:**
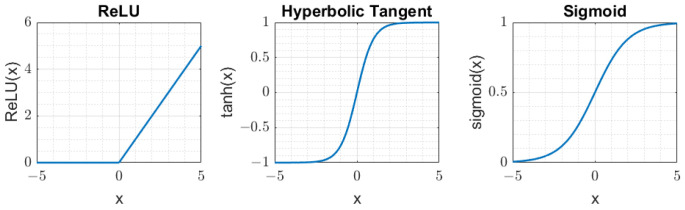
Activation functions: ReLU, tanh, and sigmoid.

Various activation functions σ can be used in the definition of an ANN, such as the hyperbolic tangent (tanh), sigmoid, or rectified linear unit (ReLU), as shown in Figure 4. The choice of the activation functions depends, among other things, on the efficiency of the resulting training algorithms used to calibrate the network weights and biases on the input and output data. For example, the ReLU is often used as an activation function in FFNNs, as the training phase is much faster than the hyperbolic tangent or sigmoid functions.

Once the number of layers and neurons and activation functions have been chosen for an NN, its weights and biases must be found (*the NN must be trained*), based on input and output data samples. An NN is trained by solving the following problem

(10a) $$\left[W^{*},b^{*}\right]=\underset{W,b}{\mathrm{argmin}}\;L\left(u,x,W,b\right),$$

(10b) $$L\left(u,x,W,b\right)=\sqrt{\frac{1}{N}\sum_{i=1}^{N}{\left(\hat{y}\left(x\right)-y\left(u\right)\right)}^{2}},$$

where *N* is the number of output samples. The cost (10b) builds on the error between the measured *y* and the predicted outputs $\hat{y}$. A solution to the problem (10a) can be found with the *backpropagation* method [29]. In backpropagation, the weights and biases are iteratively updated [30] through a standard Newton’s iteration

(11) $$\left[\begin{array}{c} W^{+}\\ b^{+} \end{array}\right]=\left[\begin{array}{c} W\\ b \end{array}\right]-\alpha \nabla L,$$

where α is the learning rate and ∇L is the *gradient* of the cost (10b). If the learning rate is large, the updated weights and bias may diverge from the optimal solution. On the other hand, a small learning rate may result in slower convergence of the learning algorithm [31]. The iterative parameters updating procedure (11) requires the initialization of the vector ${\left[W\;b\right]}^{T}$. Such initialization is crucial as it may result in a learning procedure converging to a local minimum. Unfortunately, there are no systematic procedures to initialize the iteration (11) such that local minima are avoided.

### 3.2. Recurrent Neural Networks

Recurrent Neural Networks can be used to model systems with memory, e.g., dynamical systems. RNNs are obtained by adding cyclical connections to feedforward neural networks. That is, previous values of the *predicted* output signal are fed into the network as in

(12) $${\hat{y}}_{t}=g_{t}\left({\hat{y}}_{t-1},{\hat{y}}_{t-2},\ldots ,x_{t},\ldots \right)$$

Alternatively, the following recursive structure [32,33] can be adopted

(13a) $$h_{t}=\sigma \left(W_{hx}x_{t}+W_{hh}h_{t-1}+b_{h}\right),$$

(13b) $${\hat{y}}_{t}=W_{yh}h_{t}+W_{yx}x_{t}+b_{y},$$

where $x_{t}$ and $h_{t}$ are the current inputs and hidden states; $b_{h}$ and $b_{y}$ are the biases; *W* are weights; and σ is the activation function, which can be a sigmoid function or tanh since they are both differentiable. While a DNN can also be used to model time series, previous values of the same feature need to be used as separate inputs, thus increasing the number of parameters since each input has separate weights. This is not the case in RNNs, where different time steps of the same feature (i.e., input) share the same weight.

The weights *W* and biases *b* in (13) are learned by optimizing the fit to a data set, as explained in Section 3.1. Such a learning process is affected by two well-known problems. The gradient of the loss function in (11) may exponentially decrease and shrink or exponentially increase and blow up. These phenomena are known as the *vanishing* and *exploding gradient* problems, respectively. An exploding gradient can be solved by *gradient clipping* [34]. The gradient is scaled down if it exceeds a predefined limit. However, gradient vanishing is more complex to solve. The Gated Recurrent Unit [35] and Long Short-Term Memory [23] methods are proven effective in preventing vanishing and exploding gradient problems. The latter is recalled in Section 3.3 since it is used for mass estimation in Section 4.

### 3.3. Long Short-Term Memory

In LSTM networks, the gradient vanishing problem is attacked at the training stage, by introducing *gates* in each cell of the network. Such gates, consisting of weights and sigmoid functions, selectively let the input signals to a cell in the LSTM network be *stored*, *outputted*, or *forgotten* [36]. The introduction of such gates allows control of the propagation of the state of each cell. As a result, it can be shown that, as opposed to classic (or “vanilla”) RNN, the gradient depends on the weights of the forget gates that, at the training stage, can be updated to avoid the gradient vanishing. However, such a mechanism does not prevent the gradient from exploding.

The structure of an LSTM cell [37] is

(14) $$f_{t}=\sigma \left(W_{fx}x_{t}+W_{fh}h_{t-1}+b_{f}\right)$$

(15) $$i_{t}=\sigma \left(W_{ix}x_{t}+W_{ih}h_{t-1}+b_{i}\right)$$

(16) $$o_{t}=\sigma \left(W_{ox}x_{t}+W_{oh}h_{t-1}+b_{o}\right)$$

(17) $$c_{t}=f_{t}⊙c_{t-1}+i_{t}⊙tanh\left(W_{cx}x_{t}+W_{ch}h_{t-1}+b_{c}\right)$$

(18) $$h_{t}=o_{t}⊙tanh\left(c_{t}\right)$$

where f,i, and *o* are the forget, input, and output gates, respectively, defined as in (14), (15), and (16), respectively. $W_{*x}$ and $W_{*h}$ can be referred to as the input and recurrent weights, respectively. σ is the sigmoid function. The cell state $c_{t}$ and hidden state $h_{t}$ are shown in (17) and (18). The symbol ⊙ denotes the element-wise multiplication. The final hidden state is used as an estimated output.

### 3.4. Training, Validation, and Testing

The design of an NN is an iterative procedure, which consists of the following phases:

1. *Design.* In this phase, a network structure is chosen in terms of the number of hidden layers, the number of units (neurons or cells based on the network architecture) in each layer, and the activation functions. The choice of the network architecture results in a set of weights and biases, W,b, to be learned.
2. *Learning.* The weights and biases W,b are found (*learned*) as the solution $W^{*},\;b^{*}$ of the optimization problem (10), where the deviation of the model output $\hat{y}$ from the system output measurements *y* is minimized, in the sense of cost (10b). In this phase, a set of *hyperparameters* [38] must be chosen to make the learning process fast and accurate. Such parameters include dropout probability, epoch number, weight initialization, batch size, and validation patience, which are explained next in this section.
3. *Testing.* The learned model $\hat{y}=\hat{f}\left(x,\;W^{*},\;b^{*}\right)$ is evaluated by comparing its output $\hat{y}$ against the output *y* of the actual system. If the model accuracy, that is the error $y-\hat{y}$, is not satisfactory in the RMS sense, then the design procedure restarts from the design phase, with a new network structure.

The training process of a neural network is affected by the range of the values of the input signals (i.e., the features). The unbalanced magnitude of the features may be a flaw in the training process, thus making some features completely or partially irrelevant. Such an undesirable issue can be avoided by *normalizing the features*.

The design and learning phases rely on two disjoint data sample sets that we refer to as the *training* and *validation* data sets. The training data set is used to build the cost function (10b) that is minimized in (10), through the iterative procedure (11) that requires an initialization. That is, an initial guess of the values of the weights and biases needs to be provided. Such an initialization has an enormous impact on the training process of the neural network.

If a network has been designed with more weights and biases than necessary, in the training phase, the “unnecessary” parameters are exploited to learn the noise, which unavoidably affects the measurements of the system output *y*. This phenomenon is known as *overfitting* and can be detected by evaluating the learned model $\hat{y}=\hat{f}\left(x,\;W^{*},\;b^{*}\right)$ on the validation data set that has not been used to build the function (10b). In the case of overfitting, the error $y-\hat{y}$, calculated on the validation data set, is much larger than the error calculated with the training data set. The detection of overfitting suggests adopting a network structure with fewer parameters at the design stage. Moreover, in order to prevent overfitting, *dropout*, which consists of temporarily and randomly removing neurons, can be used [39]. In dropout, with a tunable probability, neurons are removed, along with all their connections, at each weight update iteration. It should be pointed out that dropout occurs during training only.

Depending on the availability of hardware, the extensiveness of the data set, and the network complexity, training a neural network on an entire data set may be prohibitive. In such a case, the data set can be partitioned into smaller equal-sized data sets, which are called *mini-batches*. In each iteration of the training process, the weights and biases of a mini-batch are updated.

To clearly describe the complexity of the training process, an *epoch number* is defined as the multiplication of the number of iterations by the number of mini-batches. Typically, a maximum epoch number is set to limit the duration of the training. The chosen maximum epoch number should be large enough to adequately train the network. As mentioned in Section 3.1, if the learning rate is small, a larger epoch number is necessary to reach the convergence. The learning rate also depends on the chosen optimization method to train the neural network. The learning rate can be a fixed value, or it can be decreased after some iterations. On the other hand, if the cost *L* in (10b) does not decrease for more than a chosen number of iterations, then training can be stopped regardless of the maximum epoch number. This training option is called *validation patience*.

In the testing phase, the learned model is tested on “fresh” test data, i.e., data that are not included in the training and validation data sets. If the error is not satisfactory, the procedures continue with a design phase in which a new network structure is chosen with different hyperparameters.

## 4. LSTM-Based Mass Estimation

The estimation of a vehicle mass is a regression problem, where the parameter to be estimated depends on the engine torque and speed, the vehicle speed and acceleration, and a number of physical parameters, as explained in Equation (6).

Min–max normalization is used to scale features before choosing the network. For example, if the minimum and maximum values of the vehicle velocity *v* are $v_{min}$ and $v_{max}$, respectively, then the normalized velocity $v_{norm}$ is calculated by normalization as shown in (19).

(19) $$\;v_{norm}=\frac{v-v_{min}}{v_{max}-v_{min}}.$$

**Figure 5:**
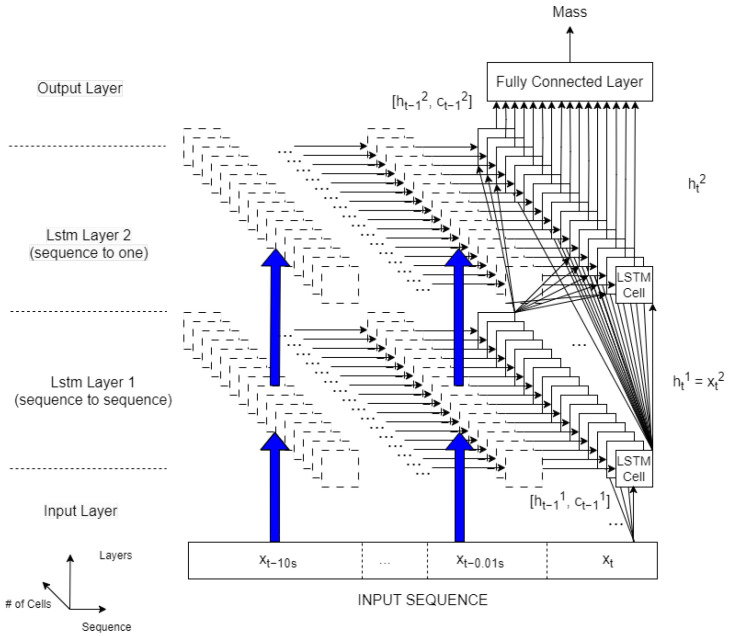
LSTM network for mass estimation.

In this study, an LSTM network is used to estimate the mass of a heavy vehicle because of its more efficient training, as compared to standard RNNs. As shown in Figure 5, the LSTM network designed in this paper has two hidden layers with 16 LSTM cells each (the rightmost solid squares in the figure). Each cell recursively processes its output signals $h_{t-i},\;c_{t-i},\;i=0,\ldots ,10$. This is sketched in Figure 5 with the dashed boxes, each visualizing the same LSTM cell processing the feature $x_{t-i}$ and its outputs $h_{t-i-1},\;c_{t-i-1}$. The output $h_{t}$ of each cell is the input of all the cells of the second layer. The two layers are referred to as *sequence-to-sequence* and *sequence-to-one* models, respectively, as the first layer outputs a sequence from a sequence while the second layer has a single output (i.e., the mass) from a sequence. In other words, in the first LSTM layer, the hidden states at each time step, which are calculated as in Equation (18), are used as the inputs of the second LSTM layer. In the second layer, hidden state and cell state information is computed at each time step as written in Equations (17) and (18). The mass is estimated by using a fully connected layer that connects the output mass with all of the hidden states of the 16 LSTM cells in the second layer at the last time step. The layer number in Figure 5 is shown by the superscripts, while the blue arrows are used to represent the same signal flow as in the rightmost part of the figure. More than 3000 parameters (weights and biases) are used to train the network. It should be noted that LSTM networks require, in general, fewer parameters to be trained than DNNs. Indeed, in DNNs, previous values of the features are additional inputs that require additional weights and biases. As a comparison, in [14], the proposed DNN for mass estimation has more than 90,000 parameters.

Such a structure is the result of a trial-and-error procedure that led to an acceptably accurate mass estimate, that is, an accuracy comparable to a commercial load sensor. Such a trial-and-error procedure is driven by the evaluation of the network made with the *validation* data set, as explained in Section 3.4.

It is worth pointing out that simpler networks (e.g., only one layer or two layers with fewer LSTM cells per layer) lead to underfitting with a lower accuracy of the estimate in the validation phase than in the accuracy goal. On the other hand, more complex LSTM networks have led to *overfitting*. That is, part of the network structure and parameters are used to learn the measurement noise. For this reason, overfitting also leads to poorly estimated mass.

In particular, a few *hyperparameters* must be set that affect the training procedure. Such hyperparameters, along with their values are shown in Table 1.

Various initialization methods can be used for starting the training phase. One of them is the *Glorot (Xavier) initializer* [41], which is a method in which the parameters to be learned are sampled from a uniform distribution between $\pm \sqrt{6/(n_{i}+n_{o})}$ where $n_{i}$ and $n_{o}$ are the numbers of inputs and outputs, respectively. Another method is *orthogonal initialization* [42], in which an orthogonal matrix Q and upper triangular matrix R are obtained from the QR decomposition of a randomly generated matrix.

Validation patience is not limited in this study, and the training process is not stopped until the end of the training. Instead, the set of computed weights and biases for training data, which results in a minimum error on the validation data, is stored whereas all other sets of weights and biases of training data that belong to other iterations are deleted.

## 5. Data Collection

Artificial data are generated by simulating, in the TruckMaker simulation environment, a truck executing a number of maneuvers. Engine torque, engine speed, longitudinal vehicle acceleration, vehicle speed, and accelerator pedal position are used as features in this study.

The training data set is built by extracting data samples from the generated artificial data, according to the following criteria.

- *Engaged clutch*. Data samples are selected if the clutch is engaged. This criterion is meant to exclude from the training data set those samples containing transients that would need extra parameters to achieve a good fit yet are unnecessary to correctly estimate the vehicle mass.
- *Fixed gear.* The training data contains data samples collected in the same gear, as training over the whole gear range would require additional parameters without increasing the mass estimation accuracy.
- *Limited speed*. Data samples are used for training for vehicle speeds in the range from 60 to 95 kph. This limitation is introduced to avoid the need for additional parameters to capture speed phenomena, e.g., wind drag, over a wide range of speeds, without increasing the estimation accuracy.
- *No braking*. The brake pedal is not used in the data samples used for training, and this feature is also eliminated since information related to braking is not as reliable as information related to propulsion in heavy-duty vehicles. The braking force resulting from a brake pedal value, e.g., may significantly vary due to the heating of the brake pads.
- *Straight driving*. Data are collected while driving straight, as only longitudinal motion is considered.
- *Flat roads.* There is neither downhill nor uphill in order not to increase complexity by adding road grade as another feature.
- *No wind.* The wind effect is neglected since there are no labeled data for wind in reality.

The data samples used for training the designed LSTM-based mass estimator are collected for nine various vehicle masses ranging from 6.8 to 7.8 tons with increments of 125 kg to curb mass. After 1-ton load is added to the curb mass of the vehicle, the procedure is repeated for a 0–2-ton load with increments of 250 kg, a 0–3-ton load with increments of 375 kg, and a 0–4-ton load with increments of 500 kg.

**Figure 6:**
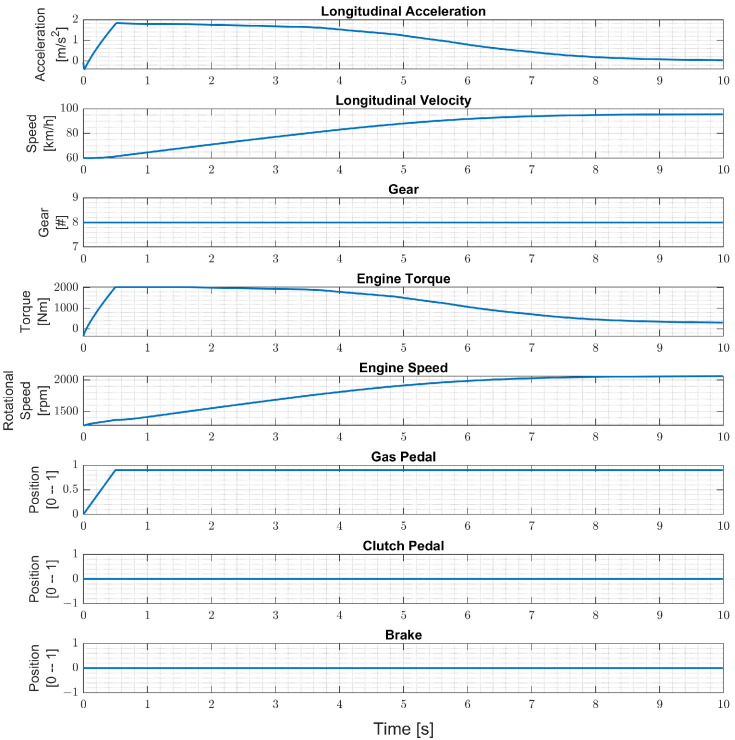
Training data with 60 kph initial velocity, 0% initial accelerator pedal percentage, and 100% final accelerator pedal percentage in 0.5 s.

Maneuvers are simulated and last 10 s each. In each simulation, an initial speed is chosen that belongs to the set {60, 70, 80, 90} kph. For each initial speed, two initial accelerator pedal position percentages are chosen, which are 0 and 100%. A total of 55 scenarios are defined for these eight combinations of the initial vehicle speed and accelerator pedal position. One of these 55 scenarios is shown in Figure 6. A total of 11 final pedal position percentages are chosen from 0 to 100% with 10% increments and times to reach these percentages that are defined as {0.5,1,2,5,10} seconds in order to have constant pedal positions and accelerations. Such combinations result in 3960 different simulations corresponding to 11 h of driving time in total for each load interval, such as 0–1 ton for a chosen truck. The validation data set consists of 495 such simulations, while the training data set contains the rest.

**Figure 7:**
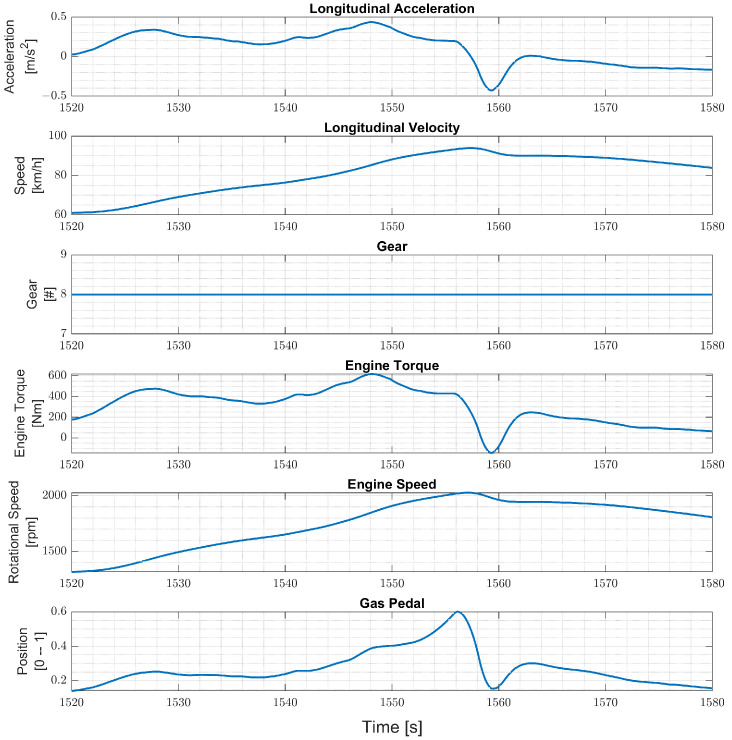
WLTC based test data in the highest gear.

TruckMaker is also used to generate the test data, which consists of data samples obtained by simulating the truck in maneuvers on a flat, straight road. A total of 21 values of masses are used by adding 200 kg to the curb mass until the maximum load is 4 tons. A vehicle speed profile is generated that resembles the Worldwide harmonized Light vehicles Test Cycle (WLTC) class 2 driving cycle. In particular, since the WLTC driving cycle is for passenger cars, in our tailored WLTC, the cycle chunks with a velocity higher than the maximum truck velocity are clipped, and the remaining cycle chunks are merged. The learned model is tested only in the highest gear. Therefore, features between 1520 s and 1580 s are used as test data with 10 s intervals. As a result, the first three tests contain only acceleration. The fourth test has both acceleration and deceleration parts, the fifth test can be accepted as a constant speed test, and the sixth test is a deceleration test, as shown in Figure 7.

To sum up, the training and validation data are collected based on excitation (various gas pedal positions and rates) whereas the test data are collected based on realistic driving cycle WLTC.

The training data set, as built in this study, requires the execution of a set of acceleration maneuvers. Such maneuvers can be extracted from the data logs of a testing vehicle, if available. Otherwise, simulation models can be used, as in this study. Another option is combining the data logs from the real truck with simulation data, which could be a viable option to reduce the training cost.

## 6. Experimental Validation

The LTSM-based vehicle mass estimation algorithm, designed as explained in Section 4, has been trained with the training and validation data sets, described in Section 5, using the MatLab R2022a Deep Learning toolbox and an NVIDIA A100 Tensor Core 40 GB GPU.

**Figure 8:**
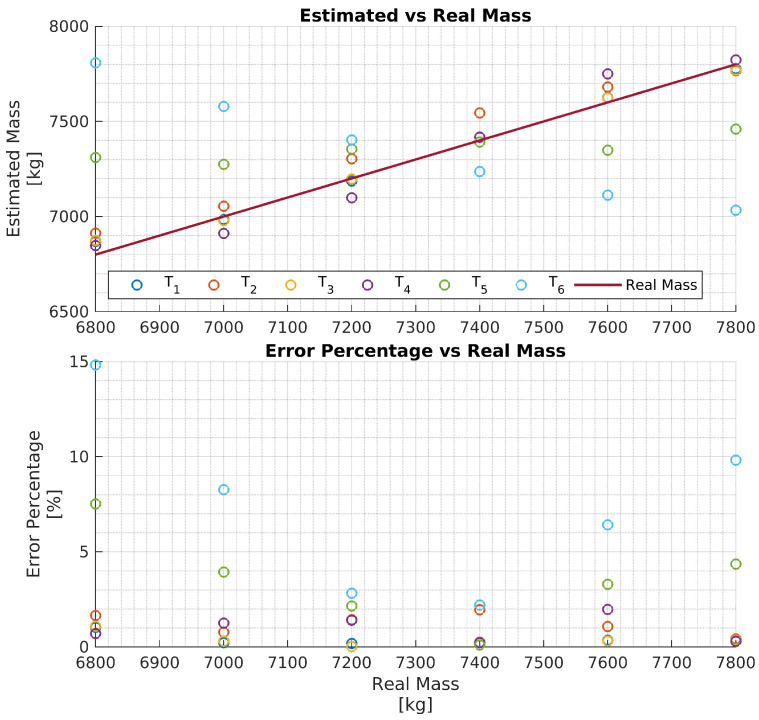
Performance of the network $N_{1}$ with the test data set.

**Figure 9:**
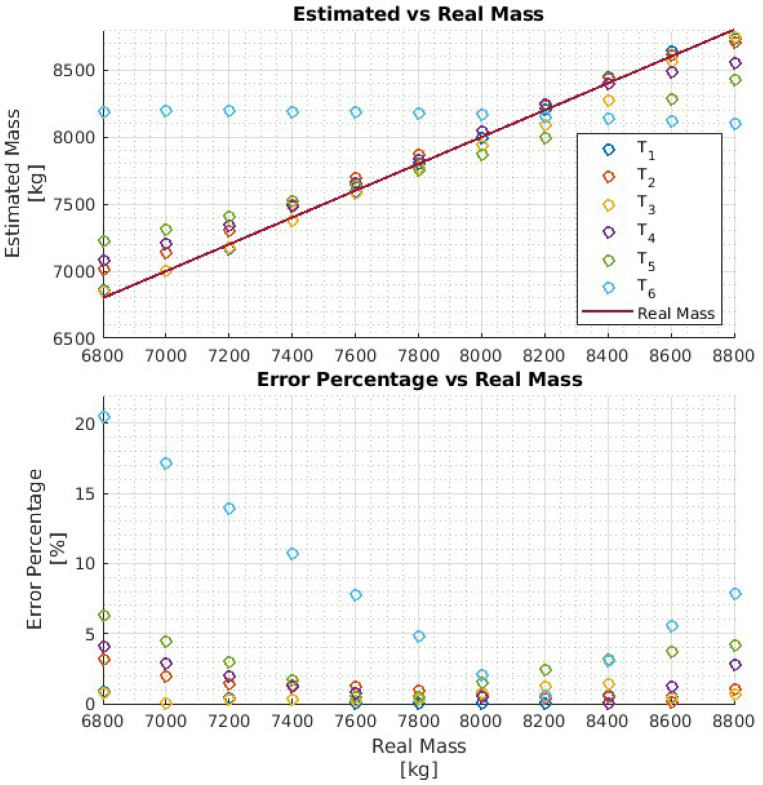
Performance of the network $N_{2}$ with the test data set.

**Figure 10:**
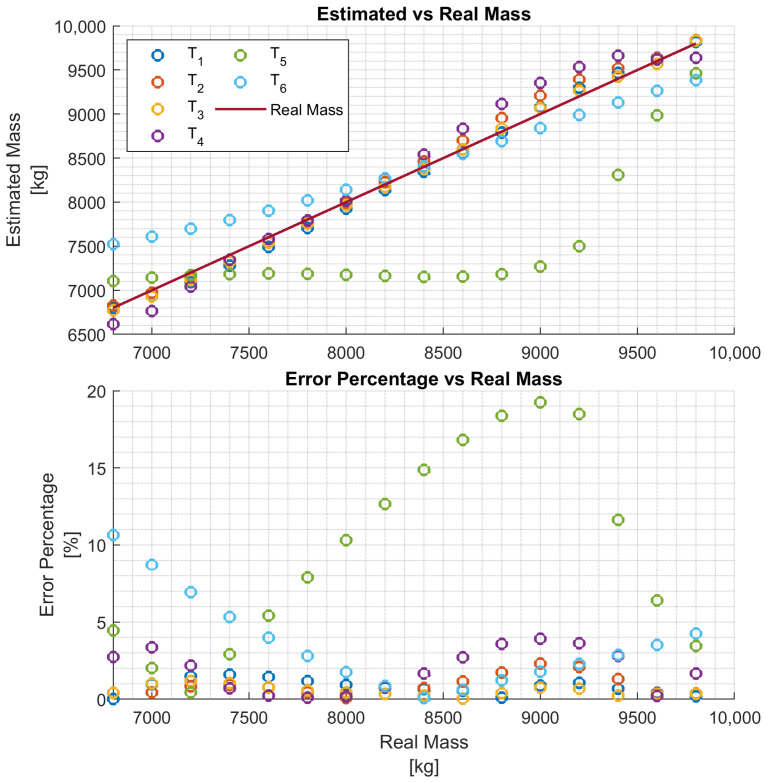
Performance of the network $N_{3}$ with the test data set.

**Figure 11:**
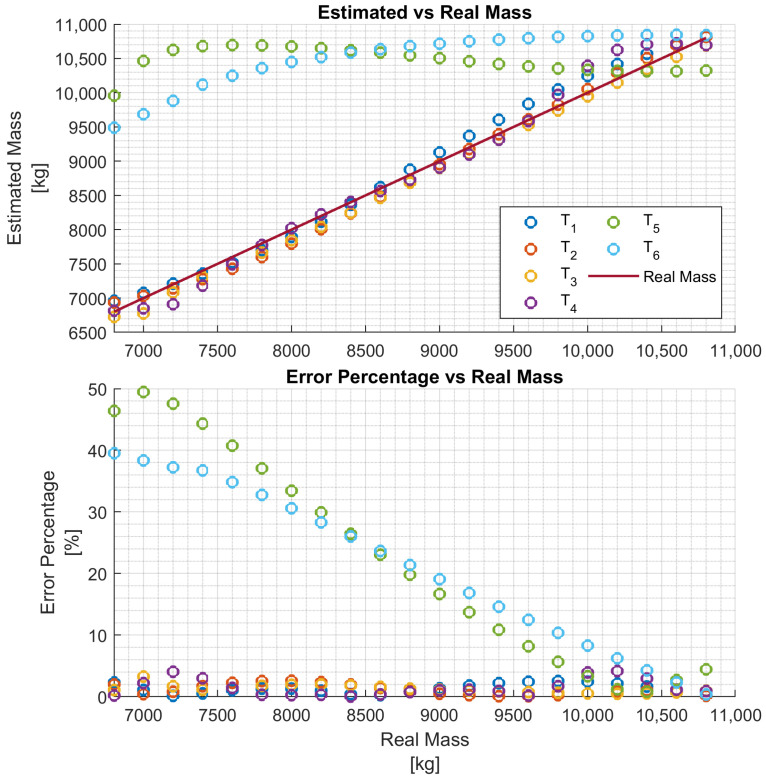
Performance of the network $N_{4}$ with the test data set.

In particular, four networks $N_{i},\;i\in \left\{1,\;2,\;3,\;4\right\}$ have been trained on four training data sets, each consisting of 11 h of driving data obtained by varying the vehicle mass in the ranges $R_{i}=\left[6.8,6.8+i\right],\;i=\left\{1,\;2,\;3,\;4\right\}$, respectively, for a chosen truck. That is, each network $N_{i}$ is trained to estimate the vehicle mass over the mass range $R_{i}$. The four networks are then evaluated with test data of the same truck, and the results are reported in Figure 8, Figure 9, Figure 10 and Figure 11, respectively. To better evaluate the performance of the four networks, the test data set has been partitioned into six 10-s time intervals $T_{j},\;j\in \left\{1,2,\ldots ,6\right\}$. For each network $N_{i}$, the maximum mass estimation error in each $T_{j}$ is reported for vehicle masses selected in the range $R_{i}$. For example, Figure 8 shows the evaluation of the network $N_{1}$ trained in the vehicle mass range $R_{1}=\left[6.8,\;7.8\right]$ tons. The six different colors show the mass estimation errors in the time intervals $T_{j},\;j\in \left\{1,2,\ldots ,6\right\}$. It can be clearly seen that the estimation errors are lower in the time intervals $T_{1},\;T_{2}$, and $T_{3}$ of the test data, corresponding to acceleration maneuvers, while the estimation error grows in $T_{4},\;T_{5}$, and $T_{6}$ where the gas pedal (the lowest plot in Figure 7) is released, and the engine brake is used. Although, in principle, training data can be generated containing more samples in the braking maneuver, this may unnecessarily decrease the accuracy of the estimate in the acceleration phase. Indeed, from the distribution of the estimation error with respect to the acceleration and/or the gas pedal signals, a very simple logic to activate the network and achieve an accurate mass estimate can be derived. Figure 9, Figure 10 and Figure 11 report the evaluation of the networks $N_{2},\;N_{3}$, and $N_{4}$ on the same test data set as in Figure 7. We recall that such networks, compared to $N_{1}$, are trained over wider mass ranges of 2, 3, and 4 tons, respectively. As expected, the estimation errors of the networks increase as the mass range is increased. Nevertheless, while all networks exhibit an estimation error below 5% in $T_{1},\;T_{2}$, and T3, in the rest of the test data set, the estimation error increases significantly. As remarked upon for the network $N_{1}$, while it could be possible to decrease the estimation error in the braking phases ($T_{4}$ to $T_{6}$), this seems to be unnecessary. As with wider ranges of vehicle mass, the networks accurately estimate the mass in acceleration maneuvers. Indeed, while it is possible, in principle, to achieve a target estimation error below 5% during a breaking maneuver, this would require a training data set that includes samples collected during braking, as well. Hence, to achieve a target accuracy in both acceleration and braking maneuvers, the size of the training data set would unavoidably increase, thus leading to a more “expensive” training phase. For a heavy-duty vehicle, whose missions very likely include docking and loading/unloading operations, it is enough to accurately estimate the mass as soon as the vehicle departs, such that the mass estimate is available to the onboard systems, e.g., the automated gear shift control system. Nevertheless, whether such a virtual load sensor actually leads to an acceptable availability of the mass estimate (with the desired accuracy) or not, should be verified with field testing.

The estimation accuracy achieved by the proposed virtual load sensor should be assessed also with respect to the available commercial sensors. The test with simulation data sets presented in this section shows that the proposed virtual sensor is a doable alternative to load sensors, which are based on leaf suspension and have an accuracy of around 5%. It must be pointed out, though, that air suspension load sensors and inclinometer-based weighing sensors exist, which have an accuracy in the range of ±2.5%.

Although the proposed virtual load sensor is as accurate as some commercial sensors and does not require any physical installation, its “training cost” can be significantly higher than the calibration costs of a hardware load sensor. For example, the LSTM network designed for the proposed virtual load sensor requires as much as 11 h of driving data. Indeed, such a data set is to be collected for each different type of powertrain with which the virtual sensors have to work, and the network also has to be retrained in the case that the network structure remains the same. Furthermore, external disturbances, such as wind, may affect the accuracy, especially at high vehicle speeds (e.g., on highways). However, this is also an issue in any model-based mass estimation method, as it is not possible to separate the effect of uncertain powertrain models and external disturbances.

## 7. Conclusions and Future Work

In this paper, a virtual load sensor is designed for heavy vehicles, based on Long Short-Term Memory neural networks. The proposed LSTM network consists of approximately 3000 parameters and achieves an estimation error within ±5% when the load varies within a 4-ton wide range. Such a network has been trained with 11 h of driving data, generated by a simulation model called Truckmaker.

As the accuracy of such an LSTM-based mass estimator decreases with the width of the load mass range, the objective of this study is to understand how the estimation error scales with the load mass range, when a fixed amount of training data (11 h of driving data in our case) is used. Our results show that, as long as the proposed LSTM-based virtual load sensor is used in accelerating maneuvers, an accuracy comparable to commercial axle load sensors can be achieved over a 4-ton wide load mass range. While such a result should be confirmed with wider (up to 10 tons) load mass ranges, this is a good starting point that helps with understanding the feasibility of such an approach with respect to the training cost (i.e., the amount of training data).

The paper also shows how the experiments can be designed to collect training and validation data that lead to a mass estimation accuracy comparable to that of commercial load sensors. The proposed approach is evaluated on the test data obtained by generating speed profiles that resemble the Worldwide harmonized Light vehicles Test Cycle (WLTC). While the data used in this paper has been generated by the high-fidelity vehicle model TruckMaker, the training and test should be repeated with the experimental data collected in experiments with real trucks.

We conclude this manuscript by recalling the most important limitations of the proposed approach when it is deployed in real vehicles. The LSTM-based load sensor presented in this paper: *(i)* as with any learning-based approach, must be re-trained for different powertrains and *(ii)* lacks accuracy in the presence of unmeasurable disturbances, e.g., wind or a snowy road surface, whose measurements are not included in the training data.

## Figures and Tables

**Table 1:** Hyperparameters and training options.

Hyperparameter	Value or Name
# of hidden layers	2
# of neurons in the hidden layers	16, 16
Output mode of LSTM layers	sequence, last
Dropout probability	0.5
Solver	adam [40]
Execution environment	gpu
# of epoch	5000
Initial learning rate	0.0025
Sequence length	longest
Output network	best-validation-loss
Gradient threshold	1

## Data Availability

The data presented in this study are available if requested from the corresponding author. The data are not publicly available due to privacy.
